# Contribution to the tribe Apalochrini (Coleoptera, Melyridae, Malachiinae) of China, with notes on the genus *Dicranolaius* Champion and *Intybia* Pascoe

**DOI:** 10.3897/BDJ.12.e139509

**Published:** 2024-11-27

**Authors:** Jia-Xiao Sun, Zhenhua Liu, Bi Ding, Rongxiang Su, Guanmin Huang, Zhiyong Xue

**Affiliations:** 1 Fujian Agriculture And Forestry University, Fuzhou, China Fujian Agriculture And Forestry University Fuzhou China; 2 Fujian Academy of Forestry, Fuzhou, China Fujian Academy of Forestry Fuzhou China; 3 Guangdong Academy of Sciences, Guangzhou, China Guangdong Academy of Sciences Guangzhou China; 4 Fujian Zhangjiangkou Mangrove National Nature Reserve Administration, Zhangzhou, China Fujian Zhangjiangkou Mangrove National Nature Reserve Administration Zhangzhou China; 5 Fugong Forestry Workstation of Longhai District Forestry Bureau, Zhangzhou, China Fugong Forestry Workstation of Longhai District Forestry Bureau Zhangzhou China

**Keywords:** soft-winged flower beetles, mangrove forests, new record, Fujian

## Abstract

**Background:**

Apalochrini is a tribe of the family Melyridae characterised by the shortened pedicel, with 13 genera recorded in China at present. *Dicranolaius* Champion and *Intybia* Pascoe of Apalochrini are distributed in Asia and Southeast Asia, with the former genus extending to Australia, but not being found in China. In a recent expedition in the mangrove forests of Zhangzhou City (Fujian Province), eastern China, specimens of *D.mangalicola* Asano ＆ Kawashima, 2010 and *I.swatowensis* (Wittmer, 1956) were collected.

**New information:**

*Dicranolaius* Champion is recorded from China for the first time and *I.swatowensis* is newly recorded for Fujian Province. Diagnostic characters of *D.mangalicola* is provided and *I.swatowensis* is re-described in details. An updated key to genera of the Chinese Apalochrini, based on male characters, is given.

## Introduction

Melyridae is a family within the superfamily Cleroidea of Coleoptera, consisting of three subfamilies, over 300 genera and 6000 species cosmopolitanly (Bocakova et al. 2012, Gimmel et al. 2019), within which, Apalochrini is a monophyletic group characterised by antennae which look 10-segmented due to the second antennomere extremely shortened and hidden by the first antennomere (Tshernyshev 2015b, Liu et al. 2024). In China, 13 genera and 45 species have been recorded, with members mostly found from waterfront habitats. Recently, a new genus, *Platyintybia* Liu et Wang, 2024, was described from south China, with a species *P.sarawakensis* (Wittmer, 1921) collected from the mangfrove forests in Zhangjiang, Fujian Province (Liu et al. 2024). Actually, another two species of Apalochrini were also collected from the same localities, which were identified to be *Dicranolaiusmangalicola* Asano & Kawashima, 2010 and *Intybiaswatowensis* (Wittmer, 1956). The former genus, *Dicranolaius* Champion is recorded from China for the first time and *I.swatowensis* is new to Fujian Province.

The genus *Dicranolaius* includes over 90 species from Oriental and Australian areas, with most species distributed on Australia, only 13 species being known from East and Southeast Asia (Asano and Kawashima 2010, Asano 2012, Liu et al. 2017, Tshernyshev 2021b). It can be recognised by the following characters in males combined: scape and antennomere 3 dilated and modified; front tarsomere 2 prolonged, bearing tarsal comb on anterior edge. *D.mangalicola* was described from Japan, inhabiting the mangrove forest (Asano and Kawashima 2010). The genus *Intybia* includes over 170 species from the Asiatic region, within which 28 species are recorded in China. It differs from *Dicranolaius* by lacking the tarsal comb in the male.

In this study, *D.mangalicola* and *I.swatowensis* are illustrated with colour images and the later species is re-described, based on the newly-collected specimens.

## Materials and methods

Materials examined in this study are deposited in the following institutions:

- **IZGAS** - Institute of Zoology, Guangdong Academy of Sciences, Guangzhou, China
- **FAF** - Fujian Academy of Forestry, Fuzhou, China.

Specimens for dissections were softened in hot water for about 10 minutes and the abdomen was removed to a glass dish with 5% solution of potassium hydroxide (KOH) for about 12 hours to clear the muscles. Dissections of male genitalia were done on open slides with glycerine on it using fine dissecting needles. Layered images were captured using the Canon 7D DSLR camera mounted on a Wemacro Focus Stacking Rail, with Canon MPE-65 mm macro lens, Mitutoyo 5× and 10× objective lens and dual-headed flash, with the help of Helicon Remote (v. 3.9.10 M) and WeMacro Control software. The images were then stacked in Helicon Focus v. 8.1.1 and edited in Photoshop CC 2022. The terminologies used in this paper follow Lawrence and Ślipiński (2013). Measurements were made with the following standards: body length-from apical edge of clypeus to apex of elytra; pronotal length-median line from anterior margin to posterior margin; pronotal width-maximum width of pronotum; elytral length-from base of scutellum to elytral apex along suture; elytral width-maximum width across the elytra.

## Taxon treatments

### Dicranolaius mangalicola

Asano＆Kawashima, 2010

D98617C6-0230-5E90-B122-7E80557F2AF7

#### Materials

- **Type status:** Other material. **Occurrence:** recordedBy: Rongxiang Su; individualCount: 1; sex: female; lifeStage: adult; occurrenceID: 4CDF178A-52C6-59A2-B85D-11440ABEE066; **Location:** country: China; stateProvince: Fujian Province; county: Zhangzhou City; municipality: Yunxiao; locality: The Zhangjiangkou Mangrove Forestry National Nature Reserve; verbatimCoordinates: 23.91843159°N, 117.42167735°E; **Identification:** identifiedBy: Zhenhua Liu; dateIdentified: 2022; **Event:** samplingProtocol: sweep net; year: 2022; month: 10; day: 17; habitat: mangrove forest; **Record Level:** basisOfRecord: PreservedSpecimen
- **Type status:** Other material. **Occurrence:** recordedBy: JiaXiao Sun; Rongxiang Su; Haitian Song; Zhiyong Xue; individualCount: 11; sex: 9 males, 2 females; lifeStage: adult; occurrenceID: E77AE6BE-127A-5379-AD89-09409995AD28; **Location:** country: China; stateProvince: Fujian Province; county: Zhangzhou City; municipality: Longhai; verbatimCoordinates: 24.39098123°N, 117.91322017°E; **Identification:** identifiedBy: Zhenhua Liu; dateIdentified: 2024-4-27; **Event:** samplingProtocol: sweep net; year: 2024; month: 4; day: 27; habitat: mangrove forest; **Record Level:** basisOfRecord: PreservedSpecimen
- **Type status:** Other material. **Occurrence:** recordedBy: Jiaxiao Sun; Rongxiang Su; Haitian Song; Guanmin Huang; individualCount: 19; sex: 8 males, 11 females; lifeStage: adult; occurrenceID: 9FF01F4D-E6E9-5842-B76E-B8C904924D34; **Location:** country: China; stateProvince: Fujian Province; county: Zhangzhou City; municipality: Yunxiao; locality: The Zhangjiangkou Mangrove Forestry National Nature Reserve; verbatimCoordinates: 23.91843159°N, 117.42167735°E; **Identification:** identifiedBy: Zhenhua Liu; dateIdentified: 2024-4-28; **Event:** samplingProtocol: sweep net; year: 2024; month: 4; day: 28; habitat: mangrove forest; **Record Level:** basisOfRecord: PreservedSpecimen

#### Diagnosis

**Figure 1.**
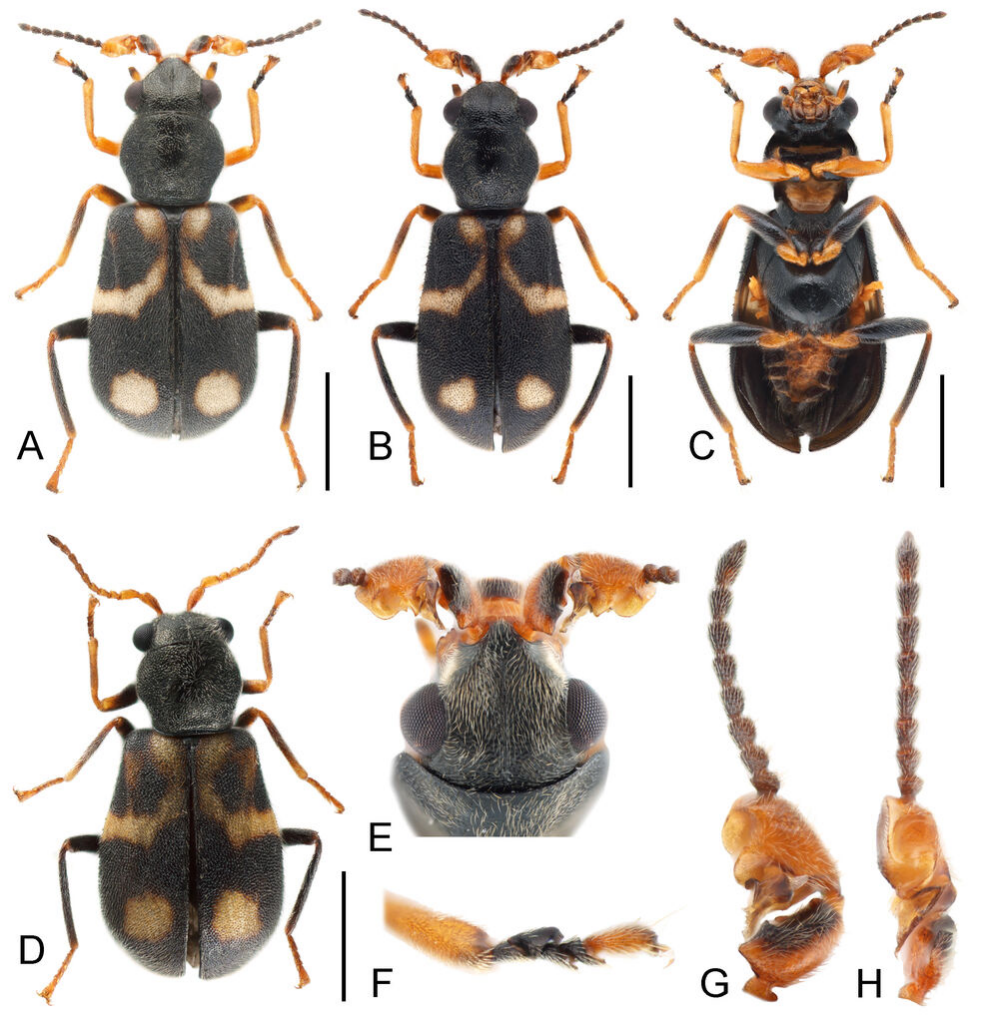
Dicranolaiusmangalicola Asano＆ Kawashima, 2010. **A** male, dorsal view; **B** male, dorsal view; **C** male, ventral view; **D** female, dorsal view; **E** head; **F** fore tarsi; **G** male antenna, dorsal view; **H** male antenna, lateral view. Scale bars: 1.0 mm (**A**-**D**).

**Figure 2.**
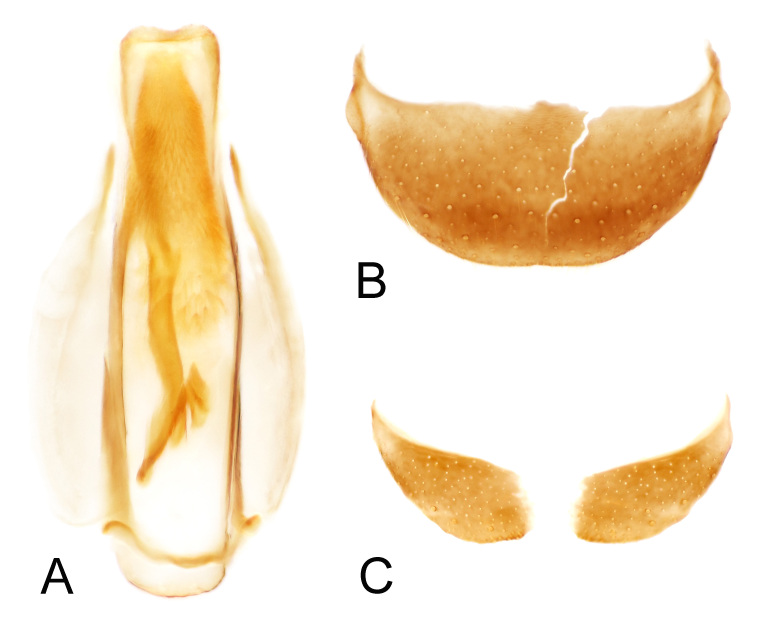
Dicranolaiusmangalicola Asano＆ Kawashima, 2010. **A** male genitalia, ventral view; **B** pygidium (apical tergite); **C** ultimate abdominal ventrite (apical sternite).

This species can be easily recognised from the congeners by colour patterns on the elytron, with a large arcuate whitish marking open to the lateral margin at base, a small yellow spot on humeral area surrounded by the arcuate marking and a large whitish spot on subapical area (Fig. 1A, B and D). Some other diagnostic characters are: head with a long median suture from vertex to frons (Fig. 1E); antennomeres 1 and 3 laterally flattened in males, scape with inner surface depressed; antennomere 3 concaved anteriorly, with a large laminate process at base (Fig. 1G and H); tergite VIII sub-trapezoid, posterior margin weakly emarginate (Fig. 2B); sternite VIII transverse, nearly divided at middle (Fig. 2C); male genitalia with median lobe sub-cylindrical, inner sac with 1 slender sclerite and 2 small spines (Fig. 2A).

#### Distribution

China: Fujian (new record for China); Japan.

#### Biology

**Figure 3.**
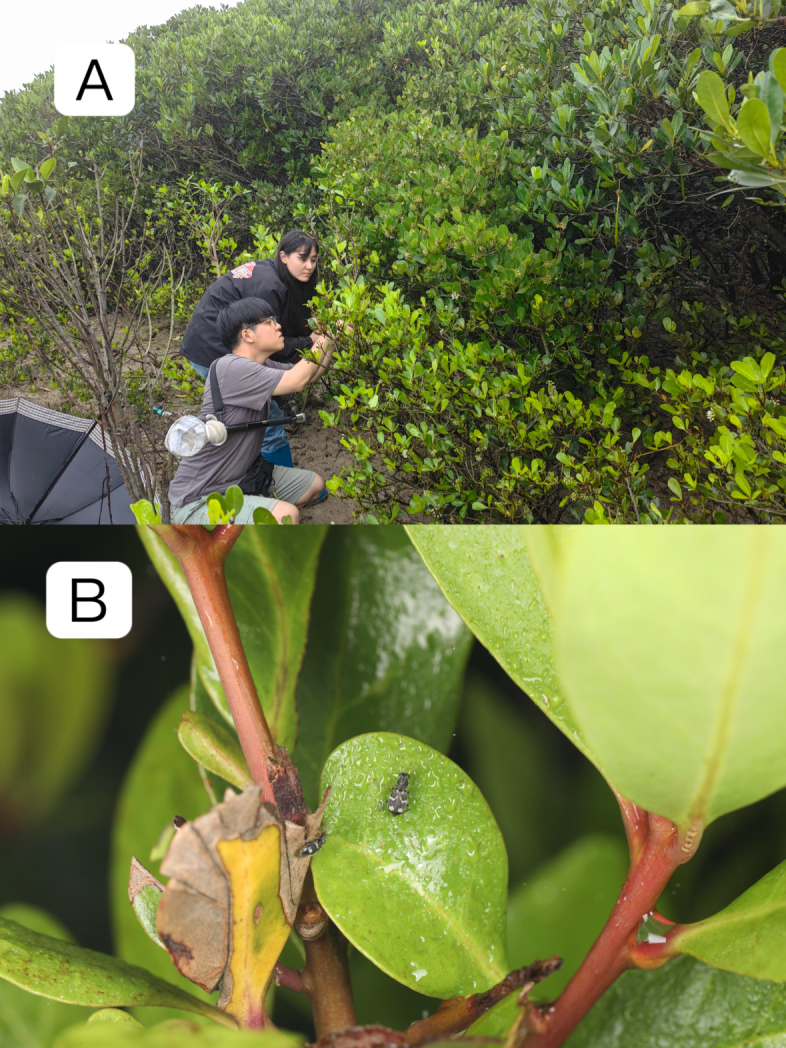
Habitat and host plant of *Dicranolaiusmangalicola* Asano＆ Kawashima, 2010. **A** habitat (photo by Haitian Song); **B** host plant *Aegicerascorniculatum* (photo by Haitian Song).

The habitat is mangrove forest (Fig. 3A), this species was collected on the leaves of *Aegicerascorniculatum* (Fig. 3B）.

### Intybia swatowensis

(Wittmer, 1956)

E906C138-D849-5625-8D9F-3F18B71F7604

#### Materials

- **Type status:** Other material. **Occurrence:** recordedBy: Rongxiang Su; individualCount: 1; sex: male; lifeStage: adult; occurrenceID: A16BB747-BCE7-5F7F-BAC4-8193590D9468; **Location:** country: China; stateProvince: Fujian Province; county: Zhangzhou City; municipality: Yunxiao; locality: The Zhangjiangkou Mangrove Forestry National Nature Reserve; verbatimCoordinates: 23.92032740°N, 117.41951987°E; **Identification:** identifiedBy: Zhenhua Liu; dateIdentified: 2022; **Event:** samplingProtocol: sweep net; year: 2022; month: 10; day: 17; habitat: mangrove forest; **Record Level:** basisOfRecord: PreservedSpecimen
- **Type status:** Other material. **Occurrence:** recordedBy: JiaXiao Sun; Haitian Song; individualCount: 2; sex: female; lifeStage: adult; occurrenceID: B7A98217-E2D9-5968-A525-309233F6FC69; **Location:** country: China; stateProvince: Fujian Province; county: Zhangzhou City; municipality: Longhai; verbatimCoordinates: 24.39098123°N, 117.91322017°E; **Identification:** identifiedBy: Zhenhua Liu; dateIdentified: 2024-4-27; **Event:** samplingProtocol: sweep net; year: 2024; month: 4; day: 27; habitat: mangrove forest; **Record Level:** basisOfRecord: PreservedSpecimen

#### Diagnosis

**Figure 4.**
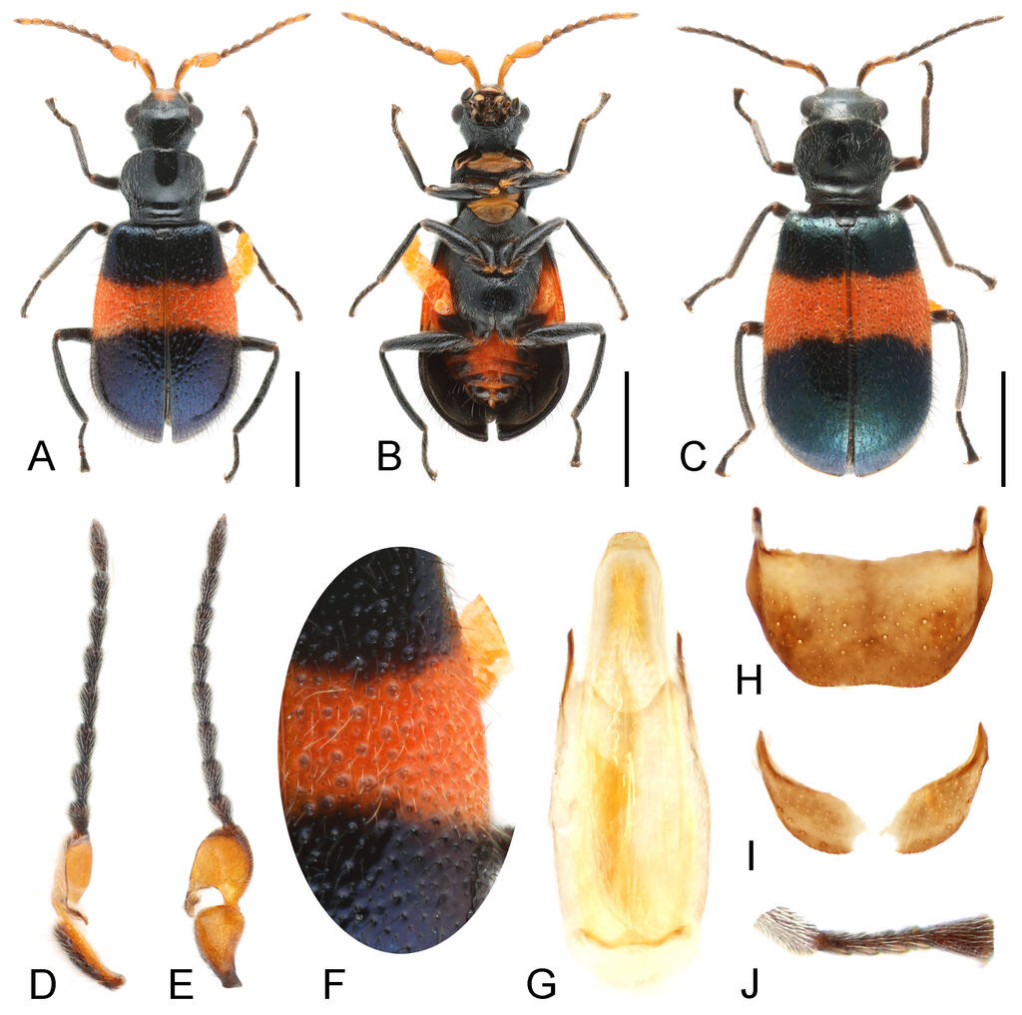
Intybiaswatowensis (Wittmer, 1956). **A** male, dorsal view; **B** male, ventral view; **C** female, dorsal view; **D** male antenna, lateral view; **E** male antenna, dorsal view; **F** elytra; **G** male genitalia, ventral view; **H** pygidium (apical tergite); **I** ultimate abdominal ventrite (apical sternite); **J** anterior tarsi, male. Scale bars: 1.0 mm (**A**-**C**).

This species can be distinguished from the other species of Intybia mainly by the shapes of antennomere 3 (Fig. 4D and E) and male genitalia (Fig. 4G); the depression behind eyes and carina next to it is also rarely known in this genus. The punctations on elytra is also distinguishable, which represented by both coarse and fine punctures mainly on central areas (Fig. 4F).

#### Distribution

China: Guangdong, Fujian (new record for Fujian).

#### Biology

**Figure 5.**
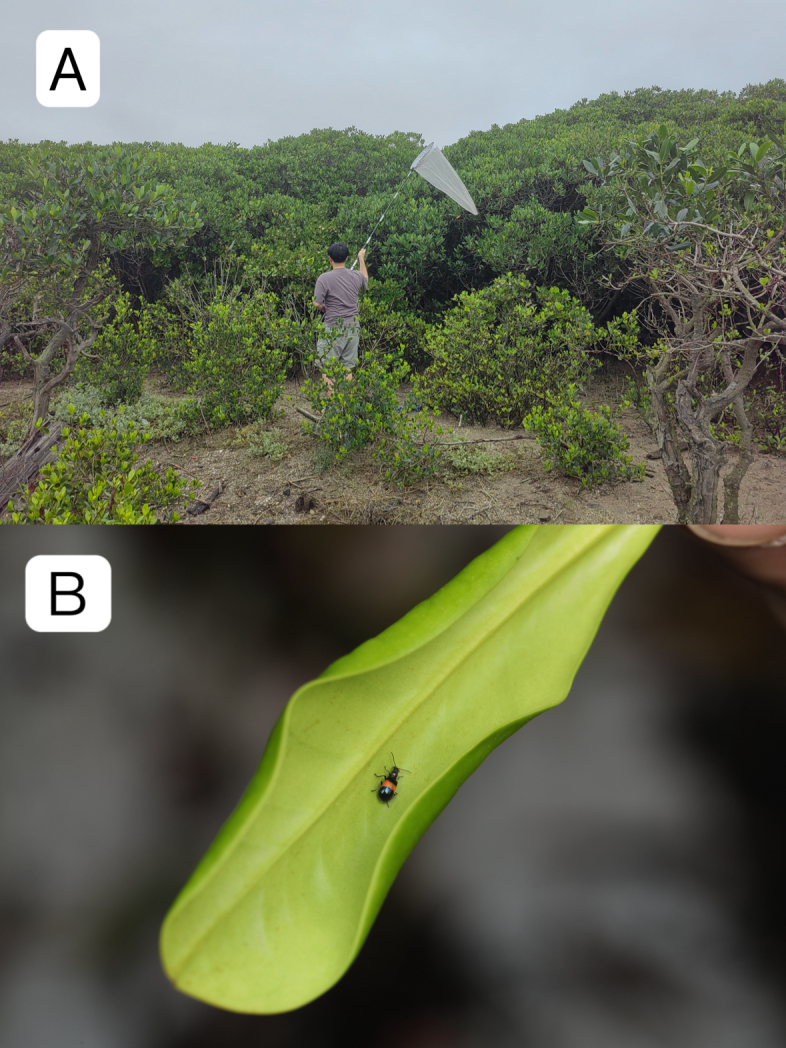
Habitat and host plant of *Intybiaswatowensis* (Wittmer, 1956). **A** habitat (photo by Haitian Song); **B** host plant *Kandeliaobovata* (photo by Haitian Song).

Specimens of *I.swatowensis* are mostly found on leaves or rocks far away from the coast (personal observation by Zhenhua Liu), but was also collected in mangrove forests in this study (Fig. 5A) and were collected on the leaves of *Kandeliaobovata* (Fig. 5B).

#### Re-description

**Male.** Length 2.9-3.5 mm. Head and prothorax black, with a quadrate orange spot on frons; antenna with basal 3 segments orange, scape with dorsal surface dark, remaining segments brown to dark brown. Elytra black with metallic bluish lustre, with an orange band across suture before middle. Legs black, anterior tarsi of male lacking comb above the 2^nd^ tarsomere. Abdomen black with median area orange. Vestiture double of dense short setae and much sparser longer bristles.

**Head.** Head narrower than pronotum; dorsal surface nearly smooth, depressed behind eyes, forming a carina from temple area to ventral side. Eyes elliptical and prominent laterally. Antenna with scape and antennomere 3 expanded and laterally flattened; scape sub-triangular with outer surface slightly depressed; antennomere 3 sub-rectangular, distinctly depressed on outer surface, with a membranous projection near base.

**Thorax.** Pronotum about 0.9 times as long as wide, lateral margins distinctly constricted at base; disc area smooth, only with a few punctations on lateral areas posteriorly, transversely depressed near base. Prosternum short, prosternal process highly reduced. Procoxal cavities large and transverse, contiguous at middle; procoxae distinctly projecting. Mesoventrite small and shield-shaped, mesepisternum and mesepimeron large. Metaventrite about as long as wide, slightly dilated. Mesocoxae triangular and projecting, meeting at middle. Metacoxae narrowly separated, abruptly narrowed to lateral. Legs relatively slender, with femora slightly enlarged near base, tarsal formula 5-5-5, anterior tarsi without comb on tarsomere 2 (Fig. 4J).

**Elytra.** Oblong-ovate, widest at about posterior third, about 1.5 times as long as wide; dorsal surface shiny, with sparse coarse punctations mostly on median areas and denser fine punctations between and next to them; vestiture mostly dark, which are yellowish on orange band.

**Abdomen.** With 6 ventrites, gradually narrowing to posterior; ventrite 1 divided at middle, only visible at lateral. Tergite VIII sub-trapezoid, with posterior margin arcuate; sternites VIII nearly divided, with median area membranous. Male genitalia with penis broadly rounded apically, inner sac with a long sclerotised flagellum and numerous tiny spines apically.

**Female.** Similar to male on body shape and colouration, but head with dorsal surface entirely black, antenna with scape and antennomere 3 only elongated.

## Identification Keys

### Updated key to genera of the Chinese Apalochrini (male only)

**Table:** 

1	Antenna with scape and antennomere 3 dilated and modified	2
–	Antenna with basal antennomeres simple	8
2	Front tarsi 4-segmented	*Platyintybia* Liu, 2024
–	Front tarsi 5-segmented	3
3	Front tarsi without comb on tarsomere 2	4
–	Front tarsi with comb on tarsomere 2	6
4	Front tibiae thickened and curved, with concavity at base	*Laius* Guérin-Méneville, 1831
–	Front tibiae simple	5
5	Body shape ant-like, with prothorax elongated and distinctly constricted posteriorly, elytra constricted at base	*Myrmecospectra* Motschulsky, 1858
–	Body shape not ant-like, prothorax never distinctly longer than wide, elytra with humeral area not distinctly constricted	*Intybia* Pascoe, 1866
6	Head with a deep hollow or protuberance, antennomere 3 dilated, but without bunch of hairs	*Troglocollops* Wittmer, 1965
–	Head with interocular area flat	7
7	Antennomere 3 dilated and with bunch of hairs	*Protocollops* Evers, 1991
–	Antennomeres 3 dilated, without bunch of hairs	*Dicranolaius* Champion，1921
8	Metathorax slightly convex, not protruding, simple and lacking appendages or tufts of hairs	9
–	Metathorax convex and bears horn-like appendage	13
9	Abdomen with aculeiform appendage on 4^th^ and 5^th^ sternites	10
–	Abdomen simple, lacking appendages	11
10	Intermediate tibiae slightly widened and excavated on inner side, anterior trochanters simple	*Opisthapalochrus* Evers, 1987
–	Intermediate tibiae simple, not widened, anterior trochanters dentate	*Spinapalochrus* Pic, 1919
11	Anterior tibiae emarginate in dorsal side, intermediate tibiae strongly dilate and convex	*Hadrocnemus* Kraatz, 1895
–	Legs simple, not emarginate or dilated	12
12	Anterior tarsi with a comb above second tarsomere. Antennae flabellate, vestiture covered with double pubescence, white and black	*Pectapalochrus* Tshernyshev, 2016
–	Anterior tarsi without a comb above second tarsomere. Antennae filiform, vestiture covered with dark pubescence	*Apalochrus* Erichson, 1840
13	Eyes extremely large, elytra impressed apically, pygidium (apical tergite) evenly rounded distally and with specifically sculptured concave-caliciform apex	*Mimapalochrus* Tshernyshev, 2015
–	Eyes and elytral apices simple, not enlarged or impressed, pygidium (apical tergite) emarginate in the middle and not sculptured	*Dromanthomorphus* Pic, 1921

(Tshernyshev 2021a, Tshernyshev 2021b)

## Discussion

Within the 51 genera of Melyridae recorded in China before this study (Major 2007, Tshernyshev 2015a, Tshernyshev 2016, Tong et al. 2022a, Tong et al. 2022b, Tong et al. 2023a, Tong et al. 2023b, Liu et al. 2023, Liu et al. 2024), 13 of them belong to the tribe Apalochrini, which is the most diverse tribe in China. The species of *Dicranolaius* found in Fujian increases the number to 14, further enriching the diversity of this group. Additionally, *D.mangalicola* together with *Platyintybiasarawakensis* (Champion, 1921) are both specific to mangrove habitats , which are not attractive to Coleopterists for their low diversity of beetles. More field works are required to investigate the distribution and diversity of those interesting beetles.

Though *Intybiaswatowensis* was also collected in mangrove forests, it is more commonly found on leaves or rocks far away from coast (personal observation by Zhenhua Liu). Thus, it is apparently not restricted to a simplex habitat as are the other two species mentioned above.

## Supplementary Material

XML Treatment for Dicranolaius mangalicola

XML Treatment for Intybia swatowensis
